# Determination of Optimal Harvest Weight for Mangalica Pigs Using a Serial Harvest Approach to Measure Growth Performance and Carcass Characteristics

**DOI:** 10.3390/foods11243958

**Published:** 2022-12-07

**Authors:** Courtney E. Charlton, Maegan Reeves Pitts, Jack G. Rehm, Jason T. Sawyer, Terry D. Brandebourg

**Affiliations:** Department of Animal Sciences, Auburn University, Auburn, AL 36849, USA

**Keywords:** Mangalica, harvest weight, growth performance, pork quality

## Abstract

Mangalica pigs are a popular niche breed given their reputation for superior pork quality. However, growth and carcass parameters for this breed are poorly documented. To better characterize optimal harvest weights for the Mangalica, a growth trial was conducted whereby pigs (n = 56) were randomly distributed across stratified harvest weights (50, 57, 68, 82, 93, 102, 127 kg) in a completely randomized design. Pigs were fed standard finisher rations with individual daily feed intakes and weekly body weights recorded for all animals. At 24 h postmortem, carcasses were split and ribbed with marbling and loin eye area (LEA) measured at the 10th rib. Primal cuts were fabricated and individually weighed. Fat back was separated from the loin and weighed. As expected, live weight significantly increased across the weight class (*p* < 0.0001). ADG was similar across classes up to 82 kg live weight, before steadily declining with increasing weight class (*p* < 0.0025). Likewise, feed efficiency did not differ between classes until weights heavier than 82 kg (*p* < 0.03). LEA significantly increased by class up to 82 kg and then plateaued as harvest weight increased further (*p <* 0.003). Marbling score significantly increased with increasing weight class up to 102 kg, where they then plateaued (*p* < 0.04). Fat back dramatically increased across all weight classes (*p* < 0.0001) despite negligible increases in LEA or marbling after 102 kg. Primal cut weights for the ham (*p <* 0.0001), loin (*p <* 0.0001), Boston butt (*p <* 0.0001), shoulder (*p <* 0.0001), and belly (*p <* 0.0001) all significantly increased with increasing live weight though significant fat deposition contributed to this gain. These data suggest an optimal harvest weight occurs between 82 to 102 kg, while offering little objective justification for harvesting Mangalica pigs at heavier live weights.

## 1. Introduction

Pork producers have responded to growing consumer demand for leaner, less fatty products by genetically selecting for pigs that grow faster, have little carcass fat, and yield more muscle at heavier harvest weights. Unfortunately, this strategy has also adversely affected important pork quality traits such as flavor, juiciness, tenderness, color, and water-holding capacity, which in turn has decreased the palatability of pork and ultimately threatens consumer demand for pork products [1,2,3]. In response to consumer perceptions of lower quality pork, niche markets have developed whereby certain consumers are willing to pay a premium price for a high-quality pork product [4]. This has renewed interest in heritage, lard-type breeds such as the Mangalica pig to meet these new market opportunities.

The Mangalica breed was first developed in Hungary during the early 1800′s and is essentially a genetically unimproved, extreme lard-type hog known for its flavorful, marbled meat cuts and especially for its high-quality lard, which can be used as an emulsifier to make excellent sausage. Mangalica lard contains more unsaturated fat than modern, improved breeds, contributing to the palatability of the meat product by providing a lower melting temperature [5]. Previous research from Roberts et al. [6] confirms that the Mangalica produces higher quality pork, as evidenced by the redder color and higher degree of marbling exhibited by this breed compared to modern breeds slaughtered at similar weights. Unfortunately, the higher propensity to fatten is associated with slower, less efficient growth, less muscle, and excessive accretion of trim or waste fat [6,7].

Generally, Mangalica producers grow pigs to very heavy harvest weights that often approach the mature size for the breed. This practice is motivated by the desire to maximize fat accumulation in growing Mangalica pigs due to the common assumption that doing so greatly improves their carcass merit. However, this assumption is not based upon empirical data. It is possible that such harvest weights are too heavy and allow carcass development to surpass a point where desirable carcass traits plateau with further gains being primarily driven by a rapidly increasing rate of fat accumulation. Unfortunately, it is not currently possible to make data-based recommendations concerning the proper harvest weight for Mangalica pigs because carcass parameters for this breed are largely uncharacterized across the Mangalica life cycle. Thus, to better allow empirical justification for the harvest weight of the breed, a growth trial was conducted in which Mangalica pigs were serially harvested across seven weight classes. Growth performance, carcass composition, and meat-quality parameters were then compared across each harvest weight class. 

## 2. Materials and Methods

### 2.1. Animals and Design

All experimental procedures were approved by the Auburn University Institutional Animal Care and Use Committee. The Auburn University College of Agriculture is accredited by the Association for Assessment and Accreditation of Laboratory Animal Care International (AALAC) and this study was conducted in accordance with the Federation of Animal Science Societies’ Guide for the Care and Use of Agricultural Animals in Research and Teaching. A total of fifty-six growing Mangalica pigs were obtained from the Auburn University research herd housed at the Auburn University Swine Research and Education Center. Pigs were individually housed in pens 12.2 m^2^ in size and were provided ad libitum access to food and water. Harvest weights were stratified into 7 weight classes (50, 57, 68, 82, 93, 102, and 127 kg live weight) spanning the traditional grow-finish stages of the porcine growth curve. The harvest weight spacing was dictated by the harvest facility schedule, with the heaviest weight class corresponding to the current industry standard harvest weight within the U.S. Pigs were randomly assigned to weight classes (n = 8) in a randomized complete block design. All pigs were fed a typical finisher ration (15% crude protein) across the 50 to 127 kg live weight classes. Daily feed intakes and weekly body weights were recorded for all animals in the test to facilitate the measurement of average daily gain (ADG), feed efficiency (kgs gained/kgs feed), dressing percentage (hot carcass weight/live weight), and total feed intake. 

### 2.2. Carcass Fabrication, Composition, and Merit Determination

Mangalica pigs were harvested at the Auburn University Lambert-Powell Meats Lab under USDA-FSIS inspection. Hot carcass weight was recorded after exsanguination and carcasses were chilled at 2 °C for 24 h, at which point, cold carcass weight was recorded. At 24 h postmortem, carcasses were split vertically to determine cold side weight and carcass length. Carcasses were then separated between the 10th and 11th rib interface where back fat (BF) depth was measured at the 1st rib, 10th rib, last rib, and last lumbar, along with the loin eye area (LEA). Primal cuts consisting of the ham, loin, Boston butt, picnic shoulder, and belly were fabricated and individually weighed and recorded along with leftover trim and fat after fabrication. Institutional Meat Purchase Specifications (IMPS) of primal cuts from the ham (IMPS# 401), loin (IMPS# 410), Boston butt (IMPS# 404), picnic shoulder (IMPS# 405), and belly (IMPS# 408) were fabricated, trimmed of external fat to an industry standard 0.635 cm, individually weighed, and recorded. The fat back was separated from the loin and weighed individually. Excess fat (fat back) on each primal was trimmed to an industry standard 0.635 cm and weighed separately from lean trimmings (trim) generated during industry-standard pork-carcass fabrication. The evaluation of subjective scores for marbling, wetness, firmness, and muscle score were determined by a trained observer using published visual standards [8]. Additionally, the longissimus muscle at the 10th rib was evaluated for objective color measurements with a Hunter Miniscan XE Plus (Hunter Lab, Reston, VA, USA) to determine Hunter L*, a*, and b* values. The Miniscan was calibrated according to the manufacturer’s recommendations and utilized a D65 light source, a 10° viewing angle, and a 35 mm viewing area. Carcass pH was recorded in the loin muscle using a pH Spear probe (Oakton Instruments, Vernon Hills, IL, USA). The pH recording is an ultimate pH measurement that occurred after carcass chilling using a temperature-compensating pH meter calibrated at two points, 4.0 and 7.0, and again after every 10 readings. 

### 2.3. Statistical Analysis

Growth performance, carcass parameters, fabricated primal cut measurements, and 24 h post-harvest pH and color scores were analyzed as a completely randomized block design using a mixed linear model of SAS v9.2 with an individual animal serving as the experimental unit, i.e., individual block and weight class serving as the main effect (SAS Institute, Inc., Cary, NC, USA). 

## 3. Results

### 3.1. Growth Performance

To better allow empirical justification of harvest weight for the Mangalica, live weight, daily feed intake, ADG, and FE were evaluated for animals on-test across the seven weight classes (Table 1). As expected, live weight significantly increased as weight class increased (*p* < 0.0001). Daily feed intake was significantly different across weight classes (*p* < 0.04); however, no apparent trend or pattern was observed. ADG (*p* < 0.0025) and FE (*p* < 0.0029) were similar across classes up to approximately 82 kg and then each steadily declined with increasing weight class. The dressing percentage significantly increased with harvest weight up to 82 kg, upon which this measure of carcass cutability began to plateau (*p* < 0.0001).

### 3.2. Longissimus Dorsi (Loin Eye) Color and pH

To better determine the ideal harvest weight for the Mangalica, visual color, objective color (L*, a*, b*), and pH were evaluated for animals on-test across the seven weight classes (Table 2). The 24 h pH was not significantly different across weight classes (*p* < 0.75) suggesting no beneficial effect on this parameter associated with harvest weight. On the other hand, visual color score progressively increased with weight class, suggesting that heavier harvest weight was associated with a more desirable redder or darker loin (*p* < 0.0032). Consistent with visual color score, the objective color measure, L* (lightness), significantly decreased as weight class increased (*p* < 0.024), while a* (redness) significantly increased as weight class increased (*p* < 0.0011); however, b* (yellowness) did not change with weight (*p* < 0.15). Thus, objective color scores also suggested that heavier harvest weights were associated with more desirable pork color.

### 3.3. Carcass Parameters and Primal Cut Measurements

To identify the appropriate harvest weight for the Mangalica, hot carcass weight, cold carcass weight, cold side weight, carcass length, and primal cuts (ham, loin, Boston butt, picnic shoulder, belly) were evaluated for animals on-test across the seven weight classes (Table 3). As expected, hot carcass weight, cold carcass weight, and cold side weight were all significantly increased in weight as the weight class increased (*p* < 0.0001). Likewise, carcass length was significantly increased with increasing weight class (*p* < 0.0001). During fabrication, the carcass is broken into characteristic primal cuts, which then give rise to the sub-primal cuts recognizable by the consumer at the meat counter. When considering these primal cuts, the ham, loin, Boston butt, picnic (shoulder), and belly all significantly increased in weight with increasing weight class (*p* < 0.0001).

### 3.4. Carcass Composition

To assess the appropriate harvest weight for the Mangalica, loin eye area, muscle score, muscle firmness, fat, fat depth along the vertebrate, marbling score, fat back (subcutaneous fat between the skin and longissimus dorsi muscle), and trim were evaluated for animals on-test across the seven weight classes (Table 4). LEA significantly increased up to 82 kg live weight and then began to plateau (*p* < 0.003). Likewise, muscle score, a subjective measure of overall leanness, increased up to 82 kg then was inconsistent without a trend (*p* < 0.0001). There were no differences in muscle firmness across weight classes (*p* < 0.08). Fat depth at the 1st, 10th, last rib, and last lumbar all significantly increased with increasing weight class (*p* < 0.0001). However, the marbling score progressively increased with increasing weight class up to 102 kg live weight where the marbling score then plateaued (*p* < 0.04). Fat back and fat both dramatically increased with increasing weight class (*p* < 0.0001). Trim was not significantly different across weight class (*p* < 0.13).

## 4. Discussion

Growth performance in the Mangalica pig is poorly characterized with few refereed manuscripts existing in the literature addressing this issue. Furthermore, the existing studies largely characterize Mangalica herds that were reared in what would be considered primitive production systems compared to modern production facilities in the U.S, often involving pasture-based systems characteristic of rural Eastern European subsistence farming. Nonetheless, a survey of such studies indicates that Mangalica pigs exhibit an ADG of 0.249 kg/day, a daily feed intake of roughly 2.3 kg/day and a feed efficiency of 0.11 [5,9,10]. In a recent study conducted at Auburn University by Roberts et al. [6] using Mangalica pigs obtained from a disease-free herd and reared in confinement while fed a concentrated ration, growth performance significantly exceeded the above performance standards. Likewise, performance by animals on-test in the current study also significantly exceeded those standards while being in general agreement with Roberts et al. [6]. Red Mangalica in the Roberts study exhibited higher marbling scores than observed in the current study (4.45 ± 0.39 at 111 kg vs. 3.42 ± 0.27 at 102 kg live weight herein). However, the contribution of several Blonde Mangalica pigs in the current trial likely lowered the overall marbling score across weight classes. Red Mangalica pigs exhibit a greater degree of marbling than Blonde Mangalica, and the Roberts study only evaluated Red Mangalica pigs [6,7]. That Mangalica growth performance in the current study exceeded levels reported in the few published studies to date is expected. Mangalica pigs on this trial and those from Roberts et al. [6] were given ad libitum access to concentrated, balanced rations formulated to match their stage of growth with the express goal of maximizing their growth rate and carcass development. This is in sharp contrast with the nutrition of Mangalica pigs in traditional growing systems in which a significant portion of their diet is met through foraging on lower quality pasture and in woodlots. Furthermore, pigs in the current study were reared in a biosecure, naïve environment (i.e., disease and parasite free), which is almost certainly not the case for pigs reared on pasture. Better growth performance would be expected from pigs that lack significant immune challenge during the growing and finishing stages.

Pork quality continues to be a serious issue in the pork industry. The emphasis on selecting pigs for leanness has resulted in a reduction in pork quality due to a loss of color and poorer marbling [11]. Color is the most important appearance quality trait affecting the visible appeal of pork to consumers [12,13,14]. Marbling is an important sensory trait that contributes to the juiciness and flavor of the product and is another key criterion impacting consumer choice at the meat counter [11]. Unfortunately, selection for leaner pigs has generally reduced the marbling content of pork, contributing to a less satisfying eating experience for the consumer [11]. This has led to the creation of niche markets whereby consumers are willing to pay a premium for high quality pork products, especially at high end restaurants [4]. Mangalica pork is currently being marketed by targeting such niche markets given the breed’s reputation for exhibiting superior pork quality and due to the higher price point necessitated by the breed’s poor growth performance. However, this reputation for superior quality is largely inferred due to the breed’s derivation, place in Eastern European cultural history, and the word of mouth of modern-day chefs. Few data exist in the literature to verify these claims. However, one recent study does support the argument that Mangalica pigs produce higher quality pork. In that study, Roberts et al. [6] directly compared Yorkshire and Red Mangalica and observed that Mangalica pork exhibited significantly higher marbling, firmness, and color scores while exhibiting lower cook loss consistent with the perception of juicier chops. Quality parameters measured in the current study were not directly compared to Yorkshire counterparts, but carcass composition and color parameter data reported herein were consistent with values observed by Roberts et al. [6]. Although the Yorkshire breed is one of the most popular breeds for pork production in the United States due to its threefold greater ADG and FE compared to heritage breeds such as the lard-type Mangalica pig, Yorkshire pork is often pale and devoid of marbling. This study, in conjunction with Roberts et al. [6], indicates that Red Mangalica exhibit darker pork with two- to threefold greater marbling scores. Clearly, regardless of rearing environment, Mangalica pigs exhibit poorer growth performance than their Yorkshire counterparts. However, these data indicate Mangalica pork displays superior meat quality attributes and suggest that the higher price points for Mangalica pork in niche markets that are needed to compensate for the poorer productive performance of this breed are indeed justified.

The current study directly addresses the wisdom of harvesting Mangalica at heavy live weights associated with the excessive accumulation of carcass fat. For instance, LEA was significantly increased up to 82 kg and then began to plateau in the current study. Primal cuts were significantly bigger as weight class increased; however, this is not a direct measure of fat-free lean meat, as this did not account for leftover subcutaneous fat along with the intermuscular and intramuscular fat of the primal cut itself. Despite increases in primal cut weight across all weight classes, LEA did not increase past 82 kg live weight. Given LEA is an excellent proxy for overall lean growth, it is very likely that a substantial proportion of primal cut weight increases that were observed across heavier weight classes was in fact due to the deposition of fat rather than lean. Unfortunately, the marbling score did not continue to increase past 102 kg live weight despite the rapid accumulation of carcass fat elsewhere. This contradicts the assumption that maximizing fat deposition on the Mangalica frame correlates to a maximal marbling score and calls into question the wisdom of utilizing harvest weights greater than 102 kg live weight. Excess adipose tissue is energetically wasteful [15] as more feed is required to support to a kg of fat accumulation than to support a similar gain in muscle. This is largely due to energy-dense lipids comprising roughly 90% of the mass of adipose tissue, whereas skeletal muscle is composed of approximately 80% water and 20% protein by weight. Thus, it is expensive to fatten animals and, after 102 kg, there is no benefit to doing so when considering either lean growth or marbling score.

Collectively, these data further support the claim that Mangalica pigs exhibit high pork quality while indicating an optimal harvest weight occurs between 82 to 102 kg depending upon the premium a producer can receive for marbling. For instance, if the producer prioritizes an optimal compromise between muscle growth and marbling, data from the present study indicate harvest at 82 kg would be ideal. On the other hand, a producer being paid a premium for marbling might harvest at 102 kg live weight as increases in marbling score were minimal beyond this weight class. These data offer little objective justification for harvesting Mangalica pigs at heavier weights unless a market is available to the producer, which provides an accessible outlet for excessive lard production.

## 5. Conclusions

Currently Mangalica producers rear their pigs to very heavy harvest weights that often approach the mature frame size for the breed. This practice is based upon a common assumption that maximizing fat accumulation on the carcass greatly improves overall carcass merit in this breed. The study herein provides the first data where carcass composition and merit were systematically examined at increasing harvest weights across the growth curve of the Mangalica pig. These data suggest that improvements in pork quality and muscle growth are modest beyond 82 kg live weight while the marbling score appears to plateau at 102 kg live weight. Meanwhile, adipose tissue accumulation increases dramatically concomitant with decreased productive efficiency across heavier live weights, resulting in an increasing cost of production with diminishing gains in carcass merit. As such, these data should allow producers to make better informed, more profitable decisions concerning the marketing of their pigs.

## Figures and Tables

**Table 1:** Growth performance of Mangalica pigs at different weight classes.

Variable ^1^	50 kg	57 kg	68 kg	82 kg	93 kg	102 kg	127 kg	SEM	*p*-Value
Final body weight, kg	50.9 ^a^	60.0 ^b^	68.9 ^c^	82.1 ^d^	92.0 ^e^	102.1 ^f^	129.8 ^g^	1.51	0.0001
Daily feed intake, kg	1.86 ^b^	1.91 ^a^	1.67 ^ab^	1.55 ^b^	1.47 ^b^	1.83 ^a^	1.84 ^a^	0.11	0.04
Average daily gain, kg	0.428 ^a^	0.500 ^a^	0.444 ^a^	0.392 ^a^	0.306 ^b^	0.315 ^b^	0.292 ^b^	0.034	0.0025
Feed efficiency ^2^	0.230 ^a^	0.260 ^a^	0.265 ^a^	0.253 ^b^	0.208 ^b^	0.172 ^b^	0.159 ^b^	0.008	0.0029
Dressing percentage	66.6 ^a^	65.5 ^a^	68.5 ^b^	71.7 ^c^	71.8 ^c^	72.8 ^c^	73.0 ^c^	0.74	0.0001

^1^ Values are group mean ± SEM, *n* = 8, differing superscripts within a variable denote differences between weigh classes, *p* < 0.05. ^2^ Feed efficiency is expressed as kg gain/kg feed intake.

**Table 2:** Loin 24 post-harvest pH and color of Mangalica pigs at different weight classes.

Variable ^1^	50 kg	57 kg	68 kg	82 kg	93 kg	102 kg	127 kg	SEM	*p*-Value
Loin Ultimate pH ^2^	5.51	5.60	5.58	5.61	5.54	5.70	5.64	0.082	0.75
Color ^3^	3.42 ^a^	3.50 ^a^	3.79 ^ab^	4.19 ^b^	3.83 ^a^	4.15 ^b^	4.33 ^b^	0.15	0.0032
L*, lightness	56.21 ^a^	57.43 ^a^	53.11 ^b^	51.58 ^a^	53.67 ^b^	52.94 ^b^	50.13 ^c^	1.34	0.024
a*, redness	9.89 ^a^	10.60 ^a^	10.73 ^a^	12.99 ^b^	11.47 ^b^	11.31 ^b^	12.79 ^b^	0.53	0.0011
b*, yellowness	14.89	16.36	14.49	16.52	15.03	14.76	14.56	0.72	0.15

^1^ Values are group mean ± SEM, *n* = 8, differing superscripts within a variable denote differences between weight classes, *p* < 0.05. ^2^ Ultimate pH: measured 24 h post-harvest on chilled carcasses. ^3^ Visual (subjective) color score: five-point scale where 1 = very light and pale; 5 = dark red etc.

**Table 3:** Carcass parameters and primal cut measurements in Mangalica pigs at different weight classes.

Variable ^1^	50 kg	57 kg	68 kg	82 kg	93 kg	102 kg	127 kg	SEM	*p*-Value
Hot carcass weight, kg	33.3 ^a^	37.3 ^b^	47.2 ^c^	50.0 ^d^	66.0 ^e^	74.3 ^f^	95.4 ^g^	1.5	0.0001
Cold carcass weight, kg	32.7 ^a^	36.7 ^b^	45.2 ^c^	57.7 ^d^	64.0 ^e^	72.4 ^f^	93.7 ^g^	1.4	0.0001
Cold side weight, kg	16.2 ^a^	18.5 ^b^	22.5 ^c^	28.3 ^d^	31.1 ^e^	35.5 ^f^	46.8 ^g^	0.8	0.0001
Carcass length, cm.	62.7 ^a^	62.7 ^a^	65.8 ^b^	70.1 ^c^	71.6 ^d^	73.2 ^e^	78.0 ^f^	0.8	0.0001
Ham, kg	3.1 ^a^	3.5 ^b^	4.3 ^c^	5.1 ^d^	4.8 ^d^	5.9 ^e^	7.3 ^f^	0.2	0.0001
Loin, kg	2.6 ^a^	3.0 ^ab^	3.4 ^b^	4.2 ^c^	4.5 ^c^	5.7 ^d^	7.0 ^d^	0.2	0.0001
Butt, kg	1.2 ^a^	1.2 ^a^	1.6 ^b^	2.2 ^c^	2.4 ^cd^	2.7 ^d^	3.6 ^e^	0.1	0.0001
Picnic, kg	1.3 ^a^	1.5 ^a^	2.0 ^b^	2.4 ^c^	2.5 ^c^	2.7 ^d^	3.7 ^e^	0.1	0.0001
Belly, kg	2.0 ^a^	2.3 ^a^	2.9 ^b^	3.5 ^c^	4.0 ^c^	4.5 ^d^	5.3 ^e^	0.2	0.0001

^1^ Values are group mean ± SEM, *n* = 8, differing superscripts within a variable denote differences between weigh classes, *p* < 0.05.

**Table 4:** Carcass composition of Mangalica pigs at different weight classes.

Variable ^1^	50 kg	57 kg	68 kg	82 kg	93 kg	102 kg	127 kg	SEM	*p*-Value
Loin eye area, cm^2^	6.40 ^a^	6.93 ^ab^	7.42 ^b^	8.89 ^c^	8.28 ^c^	8.81 ^c^	8.74 ^c^	0.43	0.003
Muscle score	1.12 ^a^	1.09 ^a^	1.42 ^b^	1.63 ^c^	1.48 ^b^	1.85 ^d^	1.26 ^a^	0.09	0.0001
Muscle Firmness ^2^	2.46	2.23	2.67	2.58	2.63	2.58	2.43	0.11	0.08
Fat	2.40 ^a^	2.93 ^a^	4.26 ^b^	5.34 ^c^	7.29 ^d^	8.01 ^e^	10.38 ^f^	0.38	0.0001
Fat depth, cm									
1st rib	5.38 ^a^	5.08 ^a^	5.69 ^a^	6.68 ^b^	6.68 ^b^	7.42 ^c^	8.99 ^d^	0.30	0.0001
10th rib	3.15 ^a^	3.43 ^ab^	3.78 ^b^	4.67 ^c^	5.49 ^d^	5.51 ^d^	7.06 ^e^	0.23	0.0001
Last rib	3.05 ^a^	3.15 ^a^	3.48 ^a^	4.17 ^b^	4.04 ^b^	4.39 ^b^	5.99 ^c^	0.33	0.0001
Last lumbar	3.66 ^a^	3.43 ^a^	3.99 ^a^	4.94 ^b^	5.08 ^b^	5.72 ^c^	6.68 ^d^	0.25	0.0001
Marbling score ^3^	1.60 ^a^	1.43 ^a^	1.85 ^a^	2.04 ^a^	2.30 ^ab^	3.42 ^c^	3.41 ^c^	0.27	0.04
Fat back, kg	1.85 ^a^	1.88 ^a^	2.51 ^b^	3.84 ^c^	4.01 ^c^	5.05 ^d^	7.19 ^e^	0.34	0.0001
Trim	3.32	2.62	3.81	3.66	4.06	4.05	4.72	0.48	0.13

^1^ Values are group mean ± SEM, *n* = 8, differing superscripts within a variable denote differences between weigh classes, *p* < 0.05. ^2^ Muscle Firmness score: measured in ½ point increments with 1 = Softest and 3 = Firmest; ^3^ Marbling Score: 1 to 2.4 = Devoid; 2.5 to 4 = Traces; 4 to 5 = Slight; etc.

## Data Availability

The data presented in this study are available on request from the corresponding author.

## References

[1] Carr T.R., Walters L.E., Whiteman J.V. (1978). Carcass Composition Changes in Growing and Finishing Swine. J. Anim. Sci..

[2] Brewer M.S., Zhu L.G., McKeith F.K. (2001). Marbling effects on quality characteristics of pork loin chops: Consumer purchase intent, visual and sensory characteristics. Meat Sci..

[3] Lebret B., Čandek-Potokar M. (2022). Review: Pork quality attributes from farm to fork. Part I. Carcass and fresh meat. Animal.

[4] Honeyman M.S., Pirog R.S., Huber G.H., Lammers P.J., Hermann J.R. (2006). The United States pork niche market phenomenon1. J. Anim. Sci..

[5] Egerszegi I., Schneider F., Rátky J., Soós F., Solti L., Manabe N., Brüssow K.-P. (2003). Comparison of Luteinizing Hormone and Steroid Hormone Secretion During the Peri- and Post-Ovulatory Periods in Mangalica and Landrace Gilts. J. Reprod. Dev..

[6] Roberts M.M. (2015). The Mangalica Pig Is a Novel Biomedical Model for Human Obesity and Its Metabolic Complications. Master’s Thesis.

[7] Hallowell H.A., Higgins K.V., Roberts M., Johnson R.M., Bayne J., Maxwell H.S., Brandebourg T., Schwartz E.H. (2021). Longitudinal Analysis of the Intestinal Microbiota in the Obese Mangalica Pig Reveals Alterations in Bacteria and Bacteriophage Populations Associated With Changes in Body Composition and Diet. Front. Cell. Infect. Microbiol..

[8] Berg E.P., NPPC (2010). Pork Composition and Quality Assessment Procedures.

[9] Brüssow K.-P., Egerszegi I., Rátky J., Soós F., Casado P.G., Tuchscherer A., Toth P. (2004). Organometric data of the reproductive tract in cycling and early pregnant Hungarian Mangalica pigs. Arch. Anim. Breed..

[10] Rátky J., Brüssow K.-P., Egerszegi I., Torner H., Schneider F., Solti L., Manabe N. (2005). Comparison of Follicular and Oocyte Development and Reproductive Hormone Secretion during the Ovulatory Period in Hungarian Native Breed, Mangalica, and Landrace Gilts. J. Reprod. Dev..

[11] Baas T.J., Mabry J.W. (1998). The Impact of Genetics on Pork Quality.

[12] Faustman C., Cassens R.G. (1991). The effect of cattle breed and muscle type on discoloration and various biochemical parameters in fresh beef. J. Anim. Sci..

[13] Cheftel J., Culioli J. (1997). Effects of high pressure on meat: A review. Meat Sci..

[14] Risvik E. (1994). Sensory properties and preferences. Meat Sci..

[15] Hausman G.J., Bergen W.G., Etherton T.D., Smith S.B. (2018). The history of adipocyte and adipose tissue research in meat animals. J. Anim. Sci..

